# The value of 3D high-resolution IR-prepared fast spoiled gradient-recalled MRI in the diagnosis of meningeal carcinomatosis involving the cranial nerves

**DOI:** 10.1186/s12880-023-01166-4

**Published:** 2023-12-11

**Authors:** Lulu Xuan, Jiafu Huang, Huikang Yin, Zehua Lu, Xiaoliang Yang, Liyue Yang, Chengjun Geng

**Affiliations:** Department of Medical Imaging, Wuxi Medical College of Anhui Medical University, 904th Hospital of Joint Logistic Support Force of PLA, Wuxi, 214044 Jiangsu China

**Keywords:** Cranial nerve, Meningeal carcinomatosis, IR-prepped, Magnetic resonance imaging, Spoiled gradient recalled

## Abstract

**Purpose:**

The purpose of this study was to investigate the clinical utility of three-dimension (3D) high-resolution inversion recovery (IR)-prepared fast spoiled gradient-recalled (SPGR) magnetic resonance imaging (MRI) in the diagnosis of cranial nerve meningeal carcinomatosis (MC).

**Methods:**

A total of 114 patients with MC from January 2015 to March 2020 were enrolled and their MRIs were analyzed retrospectively. All patients underwent MRIs before being administered a contrast agent. Both a 2D conventional MRI sequence and a 3D IR-prepared fast SPGR high-resolution T1-weighted (BRAVO) scan sequence were measured after contrast agent administration. The characteristics of MC and the involved cranial nerves were then examined.

**Results:**

Among the 114 MC patients, 81 (71.05%) had cranial nerve enhancement on contrast-enhanced 3D-BRAVO imaging, while only 41 (35.96%) had image enhancement on conventional MRI. The contrast-enhanced 3D-BRAVO displayed stronger image contrast enhancement of the cranial nerves than the conventional MRI (*P* < 0.001). Furthermore, detection rates for the facial and auditory nerves, trigeminal nerve, oculomotor nerve, sublingual nerve, optic nerve, glossopharyngeal/vagal/accessory nerve, and abductor nerve on contrast-enhanced 3D-BRAVO imaging were 58.77%, 47.37%, 9.65%, 8.77%, 5.26%, 3.51%, and 0.88%, respectively. We found a statistically significant difference between the affected facial and auditory nerves, as well as the trigeminal nerve, oculomotor nerve, sublingual nerve, and optic nerve.

**Conclusion:**

In MC, contrast-enhanced 3D-BRAVO imaging displayed the cranial nerves more effectively than 2D conventional enhanced MRI. The facial, auditory, and trigeminal nerves are the primary nerves involved in MC, and improved scanning of these nerves would aid in the early detection and treatment of MC.

## Introduction

Meningeal carcinomatosis (MC) is a severe condition caused by the spread of malignant cells to the meninges [1, 2]. The lack of specificity of the clinical indications, which are characterized by headaches, cognitive impairments, and the possibility of developing localized lesions [3], makes it challenging to diagnose MC. It has been reported that the earliest sign of MC is cranial nerve palsy, which includes facial nerve palsy and hearing loss [4, 5]. Due to factors such as longer life expectancy, improved diagnostic imaging, and the brain’s resistance to chemotherapy, there have been an increasing number of reports of MC [3]. Early detection and diagnosis of lesions are beneficial for the timely treatment of patients with meningeal cancer to improve their quality of life and prognosis. Although repeated lumbar punctures and cerebrospinal fluid (CSF) cytology are sometimes required [6], these techniques are regarded as the gold standard for diagnosing this disease [7]. CSF examination is limited in its ability to detect malignant lesions on the meninges, but imaging examinations have distinct advantages for detecting meningeal lesions.

Conventional two-dimension (2D) T1-weighted imaging (T1WI) magnetic resonance imaging (MRI) with contrast agent administration is primarily utilized in clinical studies to diagnose meningeal lesions. The involvement of cranial nerves in MC using conventional T1WI with contrast enhancement typically involves the 2D acquisition with high layer thickness and low spatial resolution, which can easily miss small lesions. Recent studies have shown that three-dimension (3D) high-resolution T1W MRI can detect small lesions associated with the facial, auditory, and hypoglossal nerves as well as infectious and neoplastic meningitis [8–12].

The IR-prepared 3D fast spoiled gradient-recalled echo T1-weighted MRI is a type of imaging sequence for measuring brain volume. It is obtained using parallel technology, which produces isotropic voxels measuring 1.2 × 1.2 × 1.2 mm^3^, resulting in high spatial resolution imaging. This sequence has notable advantages over conventional 2D MRI: the obtained volume data can be reconstructed in any direction and observed from multiple angles at any point; it is a thin-layer, no-interval volume scanning, high signal-to-noise ratio sequence that is conducive to the display of small lesions, thereby decreasing the missed diagnosis rate and providing multireference information in the clinic [13].

Therefore, the purpose of this study was to evaluate the use of 3D fast spoiled gradient-recalled echo T1W MRI in the diagnosis of MC involving cranial nerves.

## Materials and methods

This study was approved by the hospital’s ethics committee and institutional review board. All patients provided written informed consent or it was provided by the parents/legal guardians if the patient was a child. A total of 114 MC patients hospitalized between January 2015 and March 2020 were examined in this study.

A diagnosis of MC required one of three National Comprehensive Cancer Network criteria: (1) the presence of tumor cells in the CSF, (2) signs and symptoms with suggestive CSF (high white blood cell count, low glucose, and high protein) in a patient known to have a malignancy, or (3) findings during a clinical examination that are consistent with MC.

In addition, the patient had to be able to tolerate magnetic resonance (MR) examinations without contraindications and have no symptoms of trauma, infectious diseases of the nervous system, or peripheral nerve diseases.

In this study, all patients underwent conventional MRI with gadolinium enhancement utilizing an advanced MRI sequence: fast spoiled gradient-recalled (SPGR) high-resolution T1-weighted sequence or a 3D inversion recovery (IR)-prepped (also referred to as the BRAVO or 3D-BRAVO sequence) on a General Electric (GE) MRI platform.

An 8-channel phased-array head coil was connected to a 3T HDx twin-gradient GE MRI scanner in order to perform the MRI scan. Axial T1-FLAIR, T2WI, sagittal T1WI, and axial T2-FLAIR sequences were among the most frequently employed conventional imaging methods. The scanning parameters were as follows for the T1-FLAIR sequence: TR = 1,800 ms, TI = 760 ms, and TE = 24, TA = 105s; for the T2-FSE sequence: TE = 96ms, TR = 7,000 ms, and echo train length (ETL) = 32, TA = 112s; and for the T2-FLAIR sequence: TI = 2,100 ms, TR = 8,002 ms, TE = 168 ms, TA = 97s. Other settings included a 256 × 256 matrix, a 1.5 mm interslice gap, a 5 mm slice thickness, and a 24 × 24 cm field of view (FOV). The injectable dose of gadobutrol used as a contrast enhancer was 0.1 mmol/kg. In addition to the sagittal, standard axial, and coronal T1-FLAIR sequences, all patients who received gadolinium injection underwent the 3D IR-prepped fast SPGR high-resolution T1-weighted sequence with the following parameters: TE = 3.0 ms; TR = 7.8 ms; TA = 161s, FOV = 24 × 24 cm; overlapping scanning interval = 0.6 mm, slice thickness = 1.2 mm, matrix = 512 × 512 cm.

In view of the delayed enhancement characteristic of intracranial lesions, 3D BRAVO enhanced scan and conventional 2D enhanced scan were performed successively after injection of contrast agent in this study.

Two senior radiologists with a combined 20 years of experience in neuroimaging evaluated the MRI scans and reached a consensus on the imaging criteria to be applied. These included the following: abnormal enhancement of meninges, changes in cranial nerve morphology, and enhancement of the cranial nerve signals. At the time of data analysis, the examiners were blinded to the imaging technique used as well as patient identity. 3D multiple planar reconstruction (MPR) and postprocessing was performed for the 3D BRAVO enhanced sequence, followed by evaluation of the reconstructed images. In the event of a disagreement, they sought consultation with another expert to reach a decision.

For abnormal enhancement of meninges, the dura-arachnoid enhancement appeared as localized or diffused, strip-like enhancement along the brain surface beneath the skull that did not penetrate deeply into the sulci. The pia meningia-subarachnoid cavity enhancement manifested as nodular and linear enhancement along the surface of the brain parenchyma, extending deep into the sulcus fissure and cistern. Mixed enhancement manifested as the simultaneous presence of the aforementioned two enhancement patterns.

**Fig. 1:**
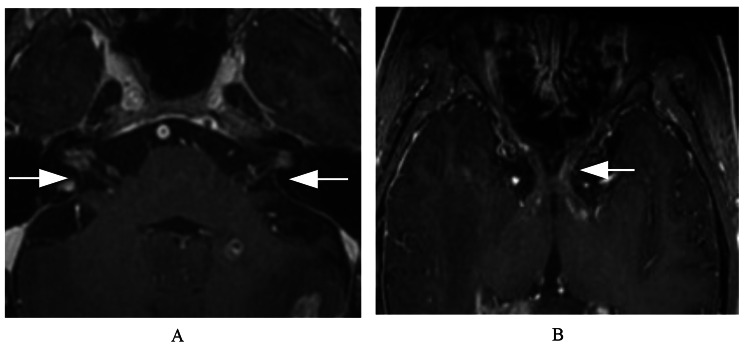
A male patient with multiple intracranial metastases of lung cancer involving the bilateral facial and auditory and optic nerves. (**A**, **B**). Contrast-enhanced BRAVO images showing nodular enhancement of nerves and strip enhancement along the nerve

**Fig. 2:**
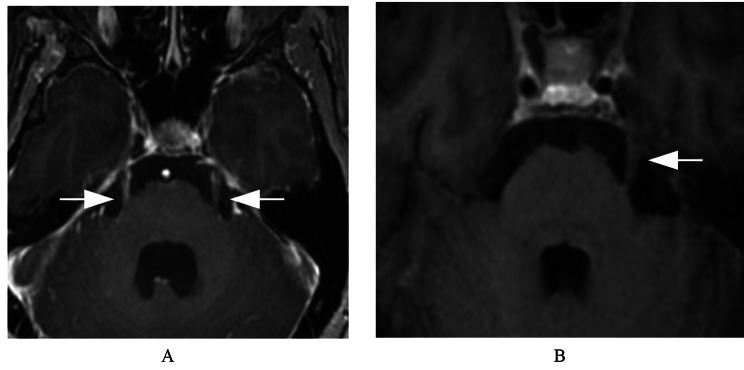
A female patient with intracranial metastasis of lung cancer involving bilateral trigeminal nerves. (**A**) Axial 3D-BRAVO shows the strip enhancement along the bilateral trigeminal nerve. (**B**) Conventional enhanced T1W image shows no abnormal enhancement in the left trigeminal nerve and the right trigeminal nerve is not seen

**Fig. 3:**
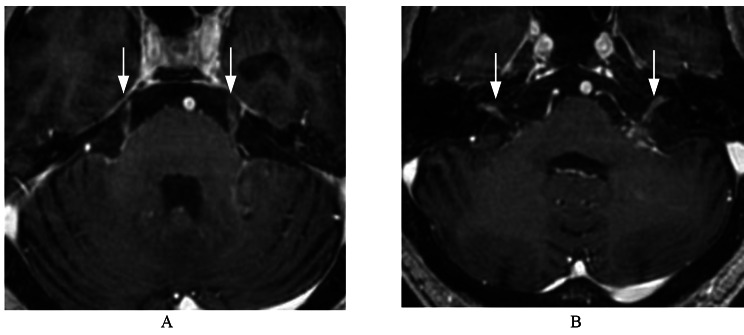
A male patient with bilateral facial and auditory nerves and trigeminal nerve involvement in intracranial metastasis from lung cancer. (**A**) Image from the contrast-enhanced BRAVO imaging showing strip enhancement along the bilateral trigeminal nerve. (**B**) Image from the contrast-enhanced BRAVO imaging showing strip enhancement along the length of the bilateral facial auditory nerves with local thickening

For abnormal enhancement of cranial nerves, the lesion often presents as a high-signal stripe or nodular enhancement along the cranial nerve, as shown in Figs. 1 and 2, and 3.

In addition, they counted the number of cases of different types of meningeal augmentation, the number and the distribution of affected cranial nerves in both the conventional MRI enhanced sequence and contrast-enhanced 3D-BRAVO imaging with image enhancement.

### Reproducibility and statistical analysis

For reproducibility analysis, twenty subjects were randomly selected for the inter-reader and intra-reader reproducibility study. The number of abnormally altered nerves was measured twice by one radiologist with a 1-month time interval to minimize the memory bias. The number of abnormally altered nerves would be measured by another radiologist for inter-reader reproducibility analysis blinded to the previous results reviewed by the first radiologist.

For statistical analysis, continuous variables are shown as mean ± standard deviation (SD) and categorical variables are presented as numbers (percentage). We counted the number of abnormally altered nerves involved and the sites with abnormal nerve changes on the left and right sides were combined (i.e., the positive findings on one side were considered to be the positive findings of the patient). Observers counted the number of positive patients in different nerve groups from the conventional 2D enhancement sequence and 3D BRAVO enhancement sequence and calculated the detection rate of affected people in different nerve groups. The Chi-squared test was used to calculate the difference between different nerve groups involved.

## Results

The cohort consisted of 67 men and 47 women ranging in age from 15 to 97 years (mean: 57.87 ± 14.80). Lung cancer comprised 55.3% of all cancer cases, followed by glioma (18.4%), breast cancer (10.8%), lymphoma (7.0%), stomach cancer (4.4%), and other malignancies (6.1%). The most prominent clinical manifestations included headache, dizziness with nausea and vomiting (n = 56, 49.1%, epilepsy and other disorders of consciousness (n = 18, 15.8%), fatigue (n = 18, 15.8%), vision loss (n = 6, 5.3%), and hearing loss (n = 3, 2.6%). Thirteen patients showed specific neurological localization dysfunction, with an incidence of 11.40%, including 5 cases of facial palsy, 3 cases of hearing loss, 2 cases of trigeminal neuralgia, 2 cases of glossopharyngeal nerve injury and 1 case of sublingual nerve injury. In addition, the relevant lesions of facial nerve, auditory nerve, trigeminal nerve, glossopharyngeal nerve and hypoglossal nerve could be found in the MRI examination images of these patients.

The affected cranial nerve involvement was observed in 81 (71.05%) patients using 3D BRAVO enhanced sequence and 41 patients (35.96%) using the conventional enhanced sequence. Using a Chi-squared test, it was determined that there was a statistically significant (*P* < 0.001) difference in detecting cranial nerve involvement between the two improved scan sequences and the baseline.

The number of branches of the affected cranial nerves is shown in Table 1. As can be seen from Table 1, the affected cranial nerves in MC patients are distributed in a variety of ways, and the facial nerves, auditory nerves (CNVII/VIII) and trigeminal nerves (CNV) are the most commonly involved parts, and the affected cranial nerves often occur simultaneously on both sides.

**Table 1:** Number of involved branches for each cranial nerve group

Cranial nerve(CN)	No. of people(n = 114)	No. of total CN	No. of affected nerve
	unilateral/bilateral		
Facial and auditory nerves	2/65	228	132(57.89%)
Trigeminal nerve	13/41	228	95(41.67%)
Oculomotor nerve	0/11	228	26(9.65%)
Hypoglossal nerve	2/8	228	18(7.89%)
Optic nerve	5/1	228	7(3.07%)
Glossopharyngeal/vagus/accessory Nerve complex	4/0	228	4(1.75%)
Abductor nerve	0/1	228	2(0.88%)

The number and proportion of patients with affected cranial nerves detected in the conventional enhanced scan sequence and the advanced sequence are shown in Table 2. As can be seen from Table 2, compared with conventional 2D enhancement sequences, 3D BRAVO enhancement sequences found more lesions in different cranial nerve groups.

**Table 2:** Number of patients with cranial nerves involved in MRI

Cranial nerve	N	Sequence		*P*
		Conventional enhanced T1-weighted	3D BRAVO enhanced sequence	
Facial and auditory nerves	114	31 (27.19%)	67 (58.77%)	< 0.001
Trigeminal nerve	114	22 (19.30%)	54 (47.37%)	< 0.001
Oculomotor nerve	114	2 (1.75%)	11 (9.65%)	< 0.001
Hypoglossal nerve	114	4 (3.51%)	10 (8.77%)	0.011
Optic nerve	114	1 (0.88%)	6 (5.26%)	0.015
Glossopharyngeal/vagus/accessory nerve complex	114	2 (1.75%)	4 (3.51%)	0.429
Abductor nerve	114	0	1 (0.88%)	1

Meningeal enhancement was observed in 97 patients (85.09%), with 12 exhibiting dural-arachnoid enhancement, 74 exhibiting pia meningia-subarachnoid cavity enhancement, and 11 exhibiting mixed enhancement. Of the 12 patients with dural-arachnoid enhancement, we found that only 1 patient developed cranial nerve lesions; Of 74 patients with pia meningia-subarachnoid cavity enhancement, 58 had cranial nerve involvement. Of the 11 patients with mixed meningeal enhancement, 7 patients developed cranial neuropathy.

In this group of cases, 17 MC patients did not have abnormal meningeal enhancement, and 16 of them showed cranial nerve involvement. In these 16 patients, a total of 49 abnormally strengthened cranial nerves were detected; however, the conventional enhanced scan detected 21 abnormal changes in cranial nerves, including 2 misdiagnosed cranial nerve lesions, which were mistaken for abnormal strengthened lesions of normal strengthened vessels.

## Discussion

Our results demonstrate that for patients with MC, the most common cranial nerve affected is CNVII, followed by CNV. The 3D-BRAVO enhanced sequence significantly improved the detection rate of cranial nerve lesions, compared with conventional 2D enhanced sequence. The 3D-BRAVO enhanced scan sequence can be reconstructed through multiple planes, tracking small blood vessels to identify lesions and strengthened vessels.

MRI with contrast enhancement is an effective technique for identifying and describing cranial nerve disorders [14]. Among the imaging symptoms of MC, atypical cranial nerve enhancement on MRI is sometimes the first or only indication of the disease process [15]. Postgadolinium sequences revealed an enlargement of the cranial nerves and an amplification of primary and secondary tumors that have progressed through the subarachnoid spaces [16].

Conventional T1WI with contrast enhancement usually uses 2D acquisition, which has a high layer thickness and poor spatial resolution, making it easy to miss small lesions. Because cranial nerves have a small and often meandering structure, they are difficult to distinguish from surrounding tissues on MR imaging [17]. The nerves also have a similar organizational structure, which makes it difficult to observe them in 2D conventional enhanced MRI and makes any abnormal enhancement easy to miss.

Because 3D BRAVO sequence has notable advantages over conventional 2D MRI, previous studies have demonstrated that the 3D BRAVO enhanced sequence can detect more and smaller lesions than conventional enhanced MRI; it can display the inner segment of the facial and hypoglossal nerves, enabling the detection of lesions [8–10]. In this study, we found that compared with conventional 2D enhanced sequence, 3D BRAVO enhanced sequence could detect more patients with cranial nerve involvement. In addition, in each group of cranial nerves, the 3D BRAVO enhanced sequence could also find more involved nerves.

In the results of this study, we can also see that the meningeal enhancement in MC patients is mainly in the form of pia meningia-subarachnoid cavity enhancement, and cranial nerves are more likely to be involved in cases of it. The reason may be related to the way of metastasis of the tumor. In this group, the primary lesions were mostly lung cancer, with a total of 63 cases, accounting for 55.26%. The main metastasis of lung cancer is blood metastasis, the pia blood supply is rich, and the probability of tumor cell invasion is greater.

The oculomotor, optic nerve, facial, auditory, trochlear, and hypoglossal nerves were the most frequently affected cranial nerves in a study of 21 patients with meningeal carcinomatosis conducted by Jin and colleagues [18]. Debnam et al. analyzed the imaging data of 270 meningeal carcinomatosis patients and found that the facial and auditory nerves and trigeminal nerve were more susceptible to involvement than other cranial nerves, accounting for 20.73% and 17.04%, respectively [19]. The incidence of imaging diagnosis of cranial nerve involvement was 71.05% among the 114 meningeal carcinomatosis patients in this study, and 13 (11.40%) had a clinical diagnosis of cranial nerve palsy. Additionally, among the affected cranial nerves, the facial, auditory, and trigeminal nerves were the most affected, which is consistent with the findings of Debnam and colleagues. The trigeminal nerve accounted for 19.30% of involvement in the conventional 2D enhanced MRI, while the facial and auditory nerves were involved in 27.19% of patients. In the 3D-BRAVO enhanced imaging, the corresponding percentages were 58.77% and 47.37%, respectively. We do note conflicting rates of incidence with the study by Debnam et al. This could be explained by the small sample size of the studies and the possibility of error in the collected data. However, the MR sequences used by Debnam et al. were conventional 2D T1WI and FLAIR sequences, both of which utilize larger layer thicknesses. It is possible that using a thin-layer 3D volume-enhanced scan could identify nerve lesions that would be missed by these methods.

According to Enzmann et al., the CSF surrounding the inner auditory canal moves slowly or forms a backflow; the facial and vestibulocochlear nerves pass through the inner auditory canal, and tumor cells are thus more likely to attach and grow on their surfaces [20]. Furthermore, the trigeminal nerve is a thick branch of the cranial nerve complex, and its large surface area is conducive to tumor cell attachment and growth. This could explain why the facial, vestibulocochlear, and trigeminal nerves are more vulnerable to involvement.

The findings of this study suggest that imaging diagnosis of cranial nerve involvement is more effective than clinical diagnosis. However, we note several limitations in this investigation. The sample size of the data is relatively small, which could introduce error. Our study only included patients whose nerve fibers had not been destroyed by the tumor cells, which were limited to the epineurium. The nerve’s function can still be compensated, but as the tumor cells continue to invade the cranial nerves and destroy the internal nerve fibers, the symptoms of cranial nerve palsy, which were initially suppressed, will eventually emerge. The majority of patients with MC seek medical treatment in a medical oncology department. Medical oncologists lack adequate physical examination training for and experience with neurology. Most MC patients experience headaches, dizziness, and vomiting, which may mask symptoms of cranial nerve palsy or the symptoms may be completely ignored or unrecognized by the patient.

## Conclusion

We report that the 3D contrast-enhanced BRAVO imaging can assist in the identification of cranial nerve invasion in meningeal carcinomatosis, which can improve the potential for timely diagnosis and treatment of clinical MC.

## Data Availability

All data are available upon request to the corresponding authors.
